# Multi-Objective Optimization of Loop Closure Detection Parameters for Indoor 2D Simultaneous Localization and Mapping

**DOI:** 10.3390/s20071906

**Published:** 2020-03-30

**Authors:** Dongxiao Han, Yuwen Li, Tao Song, Zhenyang Liu

**Affiliations:** 1 Shanghai Key Laboratory of Intelligent Manufacturing and Robotics, School of Mechatronic Engineering and Automation, Shanghai University, Shanghai 201900, China; handongxiao@shu.edu.cn (D.H.); songtao43467226@shu.edu.cn (T.S.); remeoo@i.shu.edu.cn (Z.L.); 2 Shanghai Robot Industrial Technology Research Institute, Shanghai 200062, China

**Keywords:** graph-based SLAM, loop closure detection, map evaluation, multi-objective optimization

## Abstract

Aiming at addressing the issues related to the tuning of loop closure detection parameters for indoor 2D graph-based simultaneous localization and mapping (SLAM), this article proposes a multi-objective optimization method for these parameters. The proposed method unifies the Karto SLAM algorithm, an efficient evaluation approach for map quality with three quantitative metrics, and a multi-objective optimization algorithm. More particularly, the evaluation metrics, i.e., the proportion of occupied grids, the number of corners and the amount of enclosed areas, can reflect the errors such as overlaps, blurring and misalignment when mapping nested loops, even in the absence of ground truth. The proposed method has been implemented and validated by testing on four datasets and two real-world environments. For all these tests, the map quality can be improved using the proposed method. Only loop closure detection parameters have been considered in this article, but the proposed evaluation metrics and optimization method have potential applications in the automatic tuning of other SLAM parameters to improve the map quality.

## 1. Introduction

As a key technology for autonomous navigation of mobile robots, simultaneous localization and mapping (SLAM) focuses on the problem of acquiring a spatial map of an environment while simultaneously localizing the robot using this map [1]. SLAM methods can be divided into two categories: filter-based and graph-based. Filter-based SLAM is mainly developed from the principle of recursive Bayesian estimation and is a problem of incremental, real-time data processing and robot pose correction. For example, the extended Kalman filter (EKF) can be used to estimate the robot location and geometric features in the environment [2]. However, due to its computational complexity and linearization treatment, the application of EKF SLAM has been limited by its scaling limitation and mapping inconsistence. To overcome some of these issues, particle filters have been proposed for SLAM by sampling from robot pose data associations, but the number of particles can grow large and the estimate can become inconsistent when mapping nested loops especially in large-scale environments [3,4,5]. On the other hand, graph-based SLAM models the map as a sparse graph with constraints corresponding to the relation between robot motion and environment measurement. By solving the sparse nonlinear optimization problem, graph-based methods can address the full SLAM problem of large-scale environments [6]. Kohlbrecher et al. [7] developed the Hector-SLAM scheme, which uses the Gauss-Newton equation to solve the nonlinear least square problem formulated for the front-end scan matching between the laser data and the map. Lu and Milios [8] proposed a graph-based optimization mathematical framework and Gutmann and Konolige [9] developed an efficient loop closure detection method to minimize the accumulated errors during mapping for the back-end process. With the development of optimization tools, open source implementation of graph-based SLAM frameworks is available nowadays. Konolige et al. [10] proposed Karto SLAM, the first open source graph-based framework accounting for the system sparsity. Also, Google’s open source solution Cartographer [11] introduces local submaps that integrate multi-sensor data and the matching strategy for loop closure detection. More literature on filter- and graph-based SLAM can be found in [12,13]. 

Compared with filter-based methods, graph-based SLAM can significantly reduce the accumulated error through back-end optimization and thus provide more consistent maps for large-scale environments, even ones with multiple nested loops. Loop closure detection is a most critical process in graph-based SLAM, which triggers the global nonlinear optimization by recognizing the revisited locations of the robot. Different approaches have been proposed for loop closure detection depending on the environments and application scenarios. The detection can be performed by processing the laser signals, for example, by matching the extracted features from single scan or submap [14,15,16,17,18,19] or by the matching of scan-to-scan or scan-to-submap [11,20]. The feature-based loop closure detection methods are suitable for large-scale environment, with efficient matching of the feature similarity, but these methods are limited to structured environments with obvious geometric characteristics. Compared with feature matching, scan-to-scan or scan-to-submap matching methods are more accurate and not affected by environmental features, but these methods usually have high computational cost. When processing the laser signals cannot provide sufficient positioning accuracy at certain locations in the map, auxiliary devices such as vision assisted sensors, RFID and magnetometer can be incorporated for loop closure detection [21,22,23,24,25]. 

The loop closure detection is essentially a process of matching similar scenes and involves a series of parameters. For example, in Karto SLAM, the search radius and the quantity of consecutive scans to be detected must be set for loop closure detection. These parameters should be adapted to specific applications and directly affect whether the global nonlinear optimization should be carried out at certain locations. The importance of parameter optimization or estimation related to scan matching or data sampling in SLAM algorithms has been demonstrated in literature. For instance, the intrinsic parameters of the inertial measurement unit (IMU) have been estimated online to improve the accuracy of robot trajectory [26]. In an Augmented Unscented Kalman Filter (AUKF)- based SLAM, the map quality can be improved by estimating the effective kinematic parameters such as wheel diameter, tread, and sensor mounting offset [27]. Also, it has been found that the flight parameters of an unmanned aerial vehicle (UAV), such as flight pattern, altitude, and ground speed, can significantly affect the point cloud formation during its SLAM process [28]. Several parameters of the particle filter-based Gmapping framework, such as the number of particles, the displacement update, and the resampling threshold, have been tested to determine the best configuration of these parameters, and it has been shown that these parameters have a strong impact on the mapping accuracy, CPU load and memory consumption in Gmapping [29,30]. All these works have shed some light on the necessity and difficulty of parameter optimization or tuning in SLAM either based on experience [29,30] or by establishing mathematical models [26,27,28]. However, few works can be found on how the parameters related to loop closure detection can affect the map quality and how to optimize these parameters for a given environment. This can become an issue in actual applications even with open source graph-based SLAM schemes. For example, in an industrial production scenario, the deployment of automatic guided vehicles (AGV) is usually done by operators without much SLAM experience and thus usually requires the assistance of algorithm engineer for manual tuning of these parameters, which accordingly influences the production cost, time and efficiency. To overcome this issue, a first question to answer is how to efficiently measure the mapping quality with different parameters in the SLAM algorithm for a specific environment. 

Several approaches for map evaluation have been published in the literature. The estimated map can be evaluated by comparing with a ground truth map, for example, by calculating the error metric according to the k-nearest neighbor concept [31], comparing the extracted features like SURF features and rooms [32], using image similarity methods [33,34] and topology graphs [35,36], or using artificial landmarks placed in the environment as ground truth positions [37]. In addition, since the ground truth trajectory is easier to obtain than the ground truth map, the errors between the ground truth trajectory and the estimated robot positions have been proposed to evaluate mapping accuracy as well [38,39,40]. Rather than relying on a global reference frame, relative poses can also be used for comparing the maps with different SLAM frameworks [41,42]. All these approaches for map evaluation are based on the comparison with ground truth data, which however are difficult to obtain in most applications. In the absence of ground truth, the general conditional random field [43] and several metrics including the proportion of occupied and free cells, the number of corners and the amount of enclosed areas of the estimated map [44] have been proposed to measure the mapping quality. These approaches provide efficient evaluation of SLAM quality since the measurement of map dimensions or robot poses is not required. The above map quality evaluation methods are summarized in Table 1.

Aiming at addressing the issue due to the complexity and difficulty in the tuning of the loop closure detection parameters that are critical for the back-end process of indoor 2D graph-based SLAM, this article presents a method for the multi-objective optimization of these parameters. The proposed method unifies Karto SLAM algorithm, an efficient evaluation approach for map quality with three quantitative metrics, and a multi-objective optimization algorithm. The main contribution of the article is to use three evaluation metrics, i.e., the proportion of occupied grids, the number of corners and the amount of enclosed areas, to optimize the SLAM parameters. These metrics can reflect the errors such as overlaps, blurring and misalignment when mapping nested loops, even in the absence of ground truth. Compared with exiting graph-based SLAM methods, the proposed method provides a means to further improve the mapping quality besides the back-end optimization process, by optimizing the loop closure detection parameters. For this purpose, we first summarize the general formulation of Karto SLAM and the detailed procedure and parameters for the loop closure detection. Then, quantitative metrics are presented for map evaluation without ground truth. With these metrics objectives, the Nondominated Sorting Genetic Algorithm-III (NSGA-III) multi-objective optimization algorithm is adopted to determine the parameters for loop closure detection. Finally, the proposed method is validated on four datasets and in two real indoor environments, respectively.

## 2. Problem Statement

The full SLAM problem is to estimate the environment map m and the motion trajectories of the robot $x_{1:T}{=\{x}_{1}{,\ldots ,x}_{T}\}$ in which T is the time index and $x_{i}$ are the robot poses at time i with i=1,2,…,T, through a series of motion control variables $u_{1:T}{=\{u}_{1}{,\ldots ,u}_{T}\}$ in which $u_{i}$ are odometry measurements at time i with i=1,2,…,T and the perceptions of the environment $\;z_{1:T}{=\{z}_{1}{,\ldots ,z}_{T}\}$ in which $z_{i}$ are the measurements at time i with i=1,2,…,T. Then, we can describe the SLAM problem as a process of estimating the posteriori as follows:

(1) $${p(x}_{1:T}{,m|z}_{1:T}{,u}_{1:T}{,x}_{0})$$

where $x_{0}$ represents the initial pose of the robot. By applying Bayes’ rule and following the Gaussian assumption, the full SLAM based on maximum likelihood estimation can be transformed into a constrained optimization problem. 

**Figure 1:**
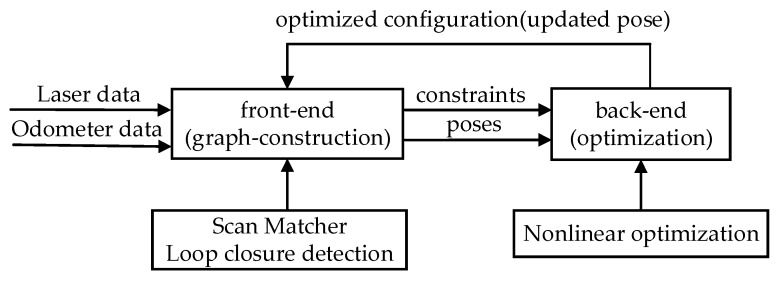
Graph-based SLAM framework [10].

The framework of graph-based SLAM is illustrated in Figure 1, which can be divided into the front-end and back-end [10]. The front-end includes scan matching to provide the estimated poses and to construct the map as a graph. The back-end is the process of nonlinear global optimization to eliminate the accumulated errors when the robot revisits the same location. Loop closure detection is a process of finding the robot itself back to the scene where it has been by scan matching and provides constraints for the back-end optimization. In Karto SLAM [10], the Sparse Pose Adjustment (SPA) is used as the back-end framework, which uses a graph to represent the history of robot measurements and each node in the graph represents the pose computed by the correlative scan matching (CSM) [45]. The edge between two nodes can be considered as a space constraint which is the measurement of node $a_{j}$ from another node $a_{i}$. This measured offset between $a_{i}$ and $a_{j}$, in the frame of $a_{i}$, is denoted as ${\bar{z}}_{ij}$. The variables $a_{i}$ are parameterized by a translational vector $t_{i}$ and a rotational angle $\theta_{i}$, in which $t_{i}={{[x}_{i}{,y}_{i}]}^{T}$ represent the robot location and $\theta_{i}$ represent its rotational angle at time i with i=1,2,3,…,T. For any two nodes $a_{i}$ and $a_{j}$, their offset can be calculated as:

(2) $$\;h\left(a_{i}{,a}_{j}\right)\equiv \left[\begin{array}{c} R_{i}^{T}{(t}_{j}{-t}_{i})\\ \theta_{j}-\theta_{i} \end{array}\right]$$

where ${h(a}_{i}{,\;a}_{j})$ represents the predicted observation, $R_{i}$ is a 2×2 rotation matrix in terms of $\theta_{i}$ as follows:

(3) $$R_{i}=\left[\begin{array}{cc} cos\left(\theta_{i}\right) & -sin\left(\theta_{i}\right)\\ sin\left(\theta_{i}\right) & cos\left(\theta_{i}\right) \end{array}\right]$$

Then the error function associated with a constraint can be written as:

(4) $$e_{ij}\equiv {\bar{z}}_{ij}-\;h\left(a_{i}{,a}_{j}\right)$$

and the total error is computed as:

(5) $$J^{2}\left(a\right)=\underset{ij}{\sum }e_{ij}^{T}\Omega_{ij}e_{ij}$$

where the information matrix $\Omega_{ij}$ is the inverse of covariance matrix between node $a_{i}$ and node $a_{j}$. Then the Levenberg-Marquardt method can be used to solve this problem by minimizing the total error in Equation (5) to determine the robot poses.

For loop closure detection, Karto SLAM algorithm generates a data chain of a certain length as a storage container for the nodes within a certain search range with respect to the current pose that are not adjacent in time, and then matches the current node with the nodes in the data chain. If the probability of matching is greater than a predefined threshold, loop closure detection is successful and the global optimization is conducted. In this process, satisfying the search range and the length of the data chain are the prerequisite for the success of loop closure detection, as well as the condition for whether the back-end optimization should be carried out. Accordingly, the parameters describing the search range and the data chain length can strongly affect the SLAM results. Usually these parameters need to be tuned by experienced SLAM engineers to improve the map quality for a specific environment. Therefore, the problem under study in this article can be stated as to determining the loop closure detection parameters, i.e., the search radius and the number of consecutive nodes in the data chain, to optimize the map quality for indoor 2D SLAM. 

## 3. Methodology

### 3.1. Loop Closure Detection

**Figure 2:**
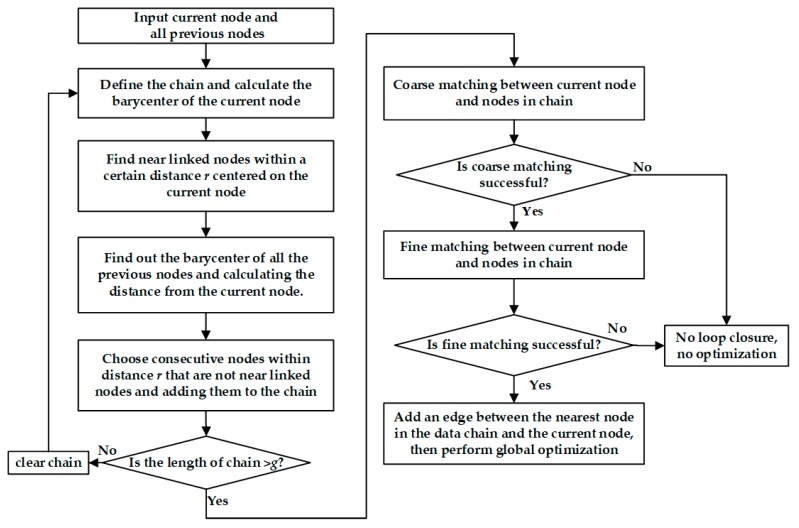
Flow chart of loop closure detection (based on the codes on GitHub Karto SLAM website).

The flow chart of the loop closure detection in Karto SLAM is illustrated in Figure 2, based on the Karto SLAM codes available on the GitHub website (https://github.com/ros-perception/slam_karto). First, several consecutive nodes of data in a certain search range are found, and then coarse matching and fine matching are performed. Once loop closure detection is successful, the corresponding relative pose is used as the constraint in the global optimization to minimize the accumulative errors in robot poses. More details on Karto SLAM can also be found in Ref. [10].

**Figure 3:**
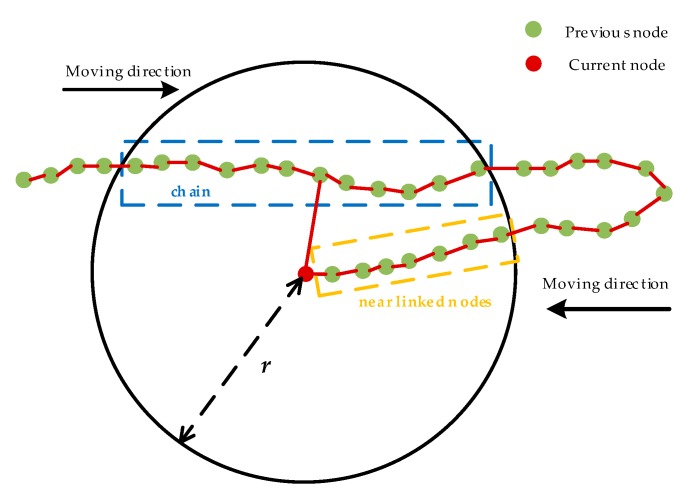
The schematic diagram of loop closure detection.

The detailed steps of loop closure detection are described as follows:

1) Take the current node as the center and find all the nodes which are connected to the current node by edges within a certain distance r using the Breadth-First Search algorithm. These nodes are called as near linked nodes that represent the latest nodes connected to the current node, as plotted in Figure 3. The red dot represents the current node, the solid green dots represent the previous nodes, and the near linked nodes are framed with an orange dotted line.
2) Calculate the distance between the current node and all the previous nodes.
3) Based on the Step 2, select all the nodes close to the current node within distance r. The loop closure detection process focuses on finding the nodes established when the robot passes through this position previously. Therefore, all the near linked nodes are excluded and the remaining nodes are added to the data chain (see the dotted blue line in Figure 3). Once the length of the data chain is larger than a predefined threshold constant g, we proceed with the matching in Step 4.
4) Perform coarse matching between the current node and the nodes in the data chain. If the resulting response value P is larger than a threshold value p and the diagonal terms in the position covariance matrix are both smaller than a certain value q, then the coarse matching is successful and perform the fine matching. Here the response value represents the environmental similarity between the current node and the nodes in the data chain found in Step 3. If the fine matching is also successful, we proceed with the global optimization in Step 5.
5) Once the loop closure detection is successful, we add an edge between the current node and the node in the data chain which is nearest to the current node (see Figure 3). Then the global optimization is performed to reduce the cumulative error.

From Figure 3, we can see that distance r represents the size of the search area for loop closure detection and g represents the quantity of consecutive nodes that have been constructed previously. Both parameters are significant in Karto SLAM because they represent the condition for whether the loop closure detection is successful to trigger the back-end optimization. 

### 3.2. Map Evaluation Metrics without Ground Truth

Even without the comparison with the ground truth, one can still evaluate a grip map by observing its image quality, for example, whether the map is skewed and whether the walls are overlapped. In this article, we evaluate the map quantitatively using the three metrics presented in [44] as the multiple objectives for the optimization of the search area *r* and the amount of nodes g for Karto SLAM. These metrics can overcome the difficulty when evaluating map quality in the absence of the ground truth, by analyzing the defects in the map such as blurring, overlaps and misalignment, due to error accumulation and lack of global optimization. It should be noted that these metrics should not be used independently to achieve more accurate results because of the contingency of a single feature.

- The proportion of occupied grids

**Figure 4:**
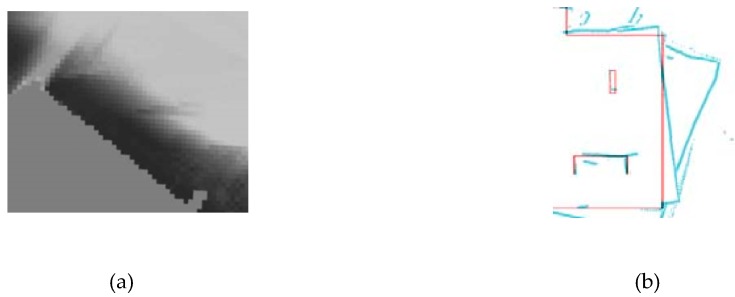
(**a**) Blurry wall [44] and (**b**) multiple occurrences wall representation [31].

The proportion of occupied grids η is defined as the number of occupied grids divided by the total number of grids in the map, i.e.:

(6) $$\eta =\frac{w_{occupied}}{w_{all}}$$

where $w_{occupied}$ represents the number of occupied grids, and $w_{all}$ represents the number of all grids in the map. When some walls or obstacles become blurred or occurred twice on the map due to the error accumulation and the loop closure detection is not satisfied, η increases. A blurry wall is shown in Figure 4a [44]; and in Figure 4b, the red wall and the blue wall represent the multiple occurrences of the same wall in the environment on the map without back-end optimization [31]. Both cases lead to an increase of the proportion of occupied grids. This indicates that a lower value of η represents a more accurate map.

- The number of corners in the map

**Figure 5:**
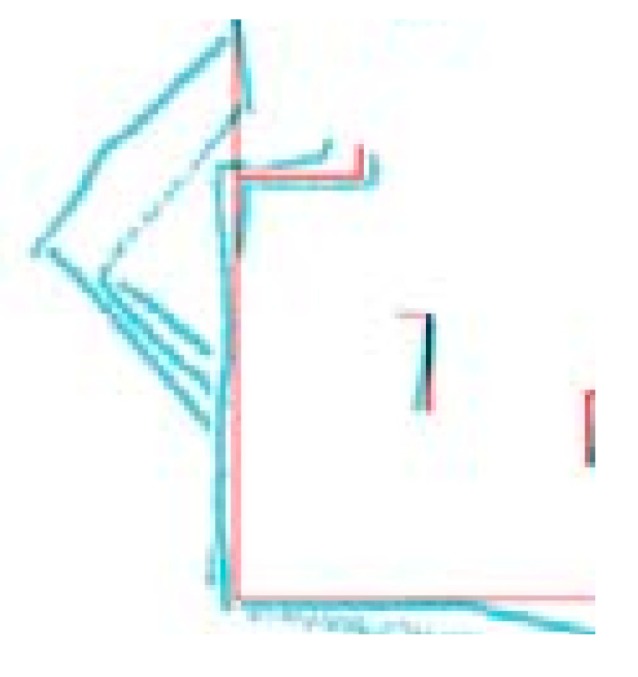
The part of a map with several corners at the same location [31].

For two maps generated by different combinations of loop closure detection parameters *r* and *g*, the map with fewer corners is more likely to be optimized successfully. As shown in Figure 5, when the robot moves through the corner, the corner is plotted in the map in red [31]. When it revisits the same corner, the map can produce another redundant corner (in blue) because of the error accumulation if the back-end optimization is not performed. Therefore, as long as no information is lost and the corners in the real world are all reflected on the grid map, the more corners the map has, the higher possibility that this map has a low quality. 

To obtain the number of corners denoted by $n_{c}$ from the map in our approach, a Gaussian filter is first applied to reduce the noise due to the environment using the *GaussianBlur* function in OpenCV. Then, the Harris corner detector [46] based on the concept of gray difference between adjacent pixel points is applied to acquire the corners on the map. To do this, a fixed window is used to slide in any direction on the image, and if there is a large gray level change in the pixel in the window after sliding, then there are corners in the window. In this article, we use the *cornerHarris* function in OpenCV to detect corners in the map.

- The amount of enclosed areas in the map

**Figure 6:**
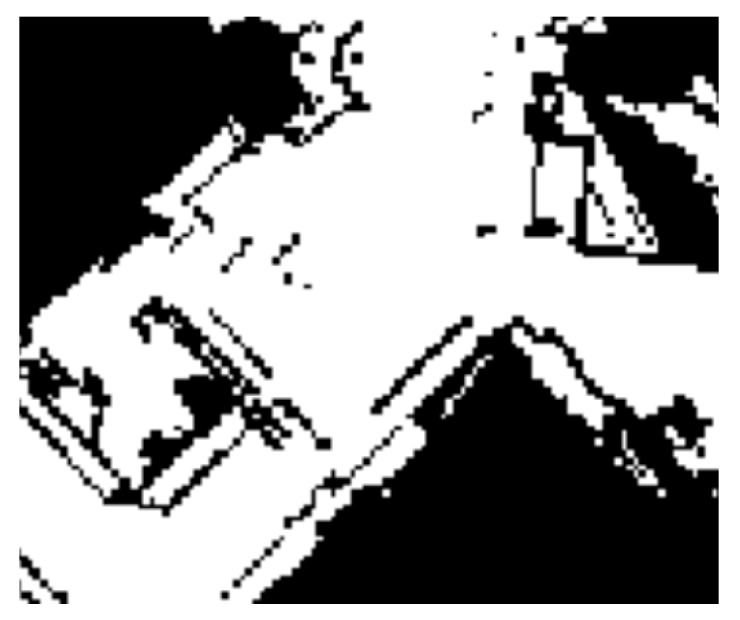
Part of a map with multiple enclosed areas.

The third metric for map evaluation is the number of enclosed areas on the map denoted by $n_{e}$. An enclosed area is a certain area completely surrounded by occupied grids on the map. This often happens in the case of error accumulation while the back-end optimization is not carried out. For example, when the same room is scanned by the robot multiple times but the loop closure detection fails, the map can contain rooms that are slightly rotated and offset to each other, resulting in multiple closed polygons on the map. An example of extracted enclosed areas is shown in Figure 6. To obtain the number of enclosed areas on the map, we first convert the occupied grid map using the *threshold* function in OpenCV to a binary image. Then, the enclosed areas can be extracted through the topological structural analysis of the binary image by border following which can be implemented through the *findContours* function in OpenCV [47].

### 3.3. NSGA-III Multi-Objective Genetic Algorithm

Evolutionary algorithms simulate the natural selection and evolution of biological organisms and have been widely used for solving complex nonlinear optimization problems. In recent years, a few evolutionary algorithms have been developed for multi-objective optimization [48,49,50,51]. Unlike many other multi-objective solving algorithms that have problems to maintain the balance of convergence and diversity, NSGA-III algorithm [48] can provide high efficiency and good performance and is suitable for solving multi-objective optimization of three dimensions and above. Therefore, NSGA-III is adopted in this article to optimize the loop closure detection parameters r and g in Karto SLAM. The multi-objective parameter optimization problem in this article can be described as:

(7) $$f\left(x\right)=min\;\{f_{1}(x),f_{2}(x),f_{3}\left(x\right)\}$$

where x=(r, g), and $f_{1}(x)=\eta \left(r,\;g\right)$, $f_{2}(x)=n_{c}\left(r,\;g\right)$, $f_{3}\left(x\right)$=$\;n_{e}\left(r,\;g\right)$.

**Figure 7:**
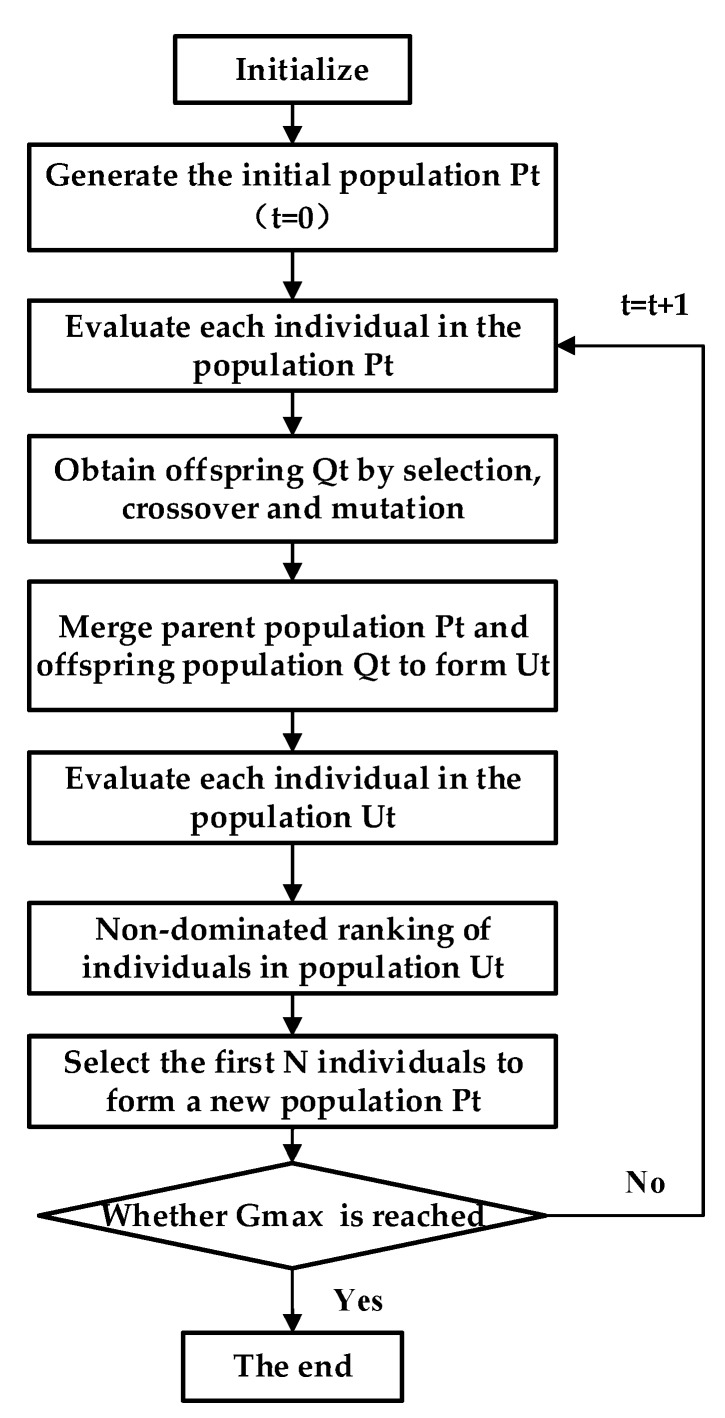
The flow chart of NSGA-III [52].

Similar to the Genetic Algorithm (GA), the parent population in NSGA-III undergoes initialization, selection, crossover, and mutation to obtain the offspring generation. Moreover, NSGA-III is a population-based heuristic algorithm, which uses a large number of well-spread reference points to maintain the diversity of the population to find a set of Pareto optimal solutions that are superior to the rest of the solutions in the search space when the multiple objectives are considered. The flow chart of NSGA-III is illustrated in Figure 7 and the detailed steps are described in the following steps [52]:

*Step 1*: Initialize parameters for the multi-objective optimization, for example, the maximum number of iterations Gmax, the population size of each generation and the ranges of the - optimization parameters.

*Step 2*: Randomly generate N individuals to form the initial population $P_{0}$. 

*Step 3*: Produce the offspring population $Q_{t}$ by the evaluation, selection, crossover and mutation of the population $P_{t}\;{(P}_{0}\;\mathrm{at}\;t=0)$, then merge the parent population $P_{t}$ and the offspring population $Q_{t}$ to form a temporary population $U_{t}$ of which the size is 2N.

*Step 4*: Carry out the non-dominant sorting of $U_{t}$ and divide $U_{t}$ to different nondomination levels $F_{i}$ with i=1,2,…,l. All the individuals from level 1 to l are first included in a set $S_{t}$.

*Step 5*: Select the first N individuals to form a new population $P_{t+1}$. According to *Step 4*, if $\left|S_{t}\right|=N$, no other operations are needed and the next generation is started with $P_{t+1}=S_{t}$. If $\left|S_{t}\right|>N$, individuals from level 1 to l−1 are selected in $S_{t}$, i.e., $P_{t+1}={\cup^{\;}}_{i=1}^{l-1}F_{i}$, and the remaining ($K=N-\left|P_{t+1}\right|$) individuals are selected from the last front $F_{l}$.

*Step 6*: Determine whether Gmax is reached. If yes, output $P_{t+1}$ as the final population; otherwise, continue with *Step 3*.

**Figure 8:**
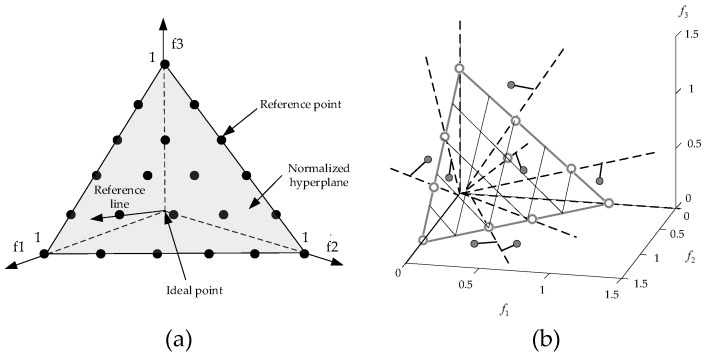
(**a**) Location of reference points and (**b**) associating individuals with reference points on a hyper-plane with M=3 and D=5.

The selection of the individuals to form a new population in *Step 5* is a most critical process in NSGA-III. By using the reference point strategy, the new population guarantees the individual diversity and the solution convergence during evolution. To do this, the reference points are first located on a normalized hyper-plane. This hyper-plane is an (*M*-1)-dimensional unit simplex equally inclined to all objective axes and it has an intercept of one on each axis, where *M* is the number of objectives. According to the normal-boundary intersection (NBI) method [51], a uniformly distributed reference point set is generated on the hyper-plane as shown in Figure 8a, with the amount of reference points can be calculated as:

(8) $$\;H=\left(\begin{array}{c} M+D-1\\ D \end{array}\right)$$

where *D* denotes the number of divisions along each axis. Then, the ideal point of $S_{t}$ is determined by identifying the minimum value of each objective, and each objective value of $S_{t}$ is adaptively normalized relative to the ideal value. Thereafter, the extreme point in each objective axis is determined by finding the individual corresponding to the minimum value of the achievement scalarizing function. A hyper-plane through these extreme points is then created. Now, a reference line is drawn by connecting the reference point and the origin point of the above hyper-plane. Each individual of $S_{t}$ is associated with the reference point, of which the reference line has the shortest perpendicular distance to that individual, as illustrated in Figure 8b. Finally, the niche-preservation operation is used to choose K individuals one at a time from $F_{l}$ to construct the population $P_{t+1}$. It should be noted that since the above-mentioned adaptive normalization of population members and the creation of the hyper-plane are performed at each generation using the extreme points, NSGA-III algorithm adaptively maintains the diversity in the space spanned by the members of $S_{t}$ at every generation. This enables NSGA-III to solve the multi-objective optimization problems with differently scaled objectives. The readers are referred to [48] for more information about NSGA-III.

## 4. Results

### 4.1. Validation on Datasets

Four publicly available datasets representing different kinds of indoor environments have been used to validate the proposed approach, i.e., the dataset of the ACES building at the University of Texas, the Austin and the Intel Research Lab dataset, the MIT Killian Court dataset, and the dataset acquired at the CSAIL at MIT. Each of these datasets has a unique sensor configuration, and the measurement data are all two-dimensional, thus we adjusted the sensor parameters in Karto SLAM algorithm according to the instructions of these datasets. The parameters involved in the proposed method in this analysis are listed in Table 2.

Note that the NSGA-III parameter values (i.e., population size, maximum number of generations) are chosen based on experience. It has been found that with the listed values, the NSGA-III algorithm can converge to the optimal solution, and using larger values of the population size and the maximum number of generations has minor influence on the optimization results while can considerably increase the computational time. The ranges of the optimization parameters are chosen as r∈ [2,6] m and g∈ [6,20] which can cover the corresponding ranges for loop closure detection for the six quite different environments tested in this article. As a result, the users of our approach do not need to determine the values of *r* and *g* for a specific environment, while NSGA-III can output their optimal values.

To demonstrate the influence of the loop closure detection parameters on the mapping results, we have used NSGA-III to find the best and worst values of r and g for each dataset for comparison, as listed in Table 3 respectively. On the other hand, little information can be found about how to tune the loop closure detection parameters *r* and *g* from the literature on Karto SLAM, such as, Refs. [10,31]. We find the default values of these two parameters in the codes provided by the GitHub website of Karto SLAM (https://github.com/ros-perception/slam_karto), i.e., r = 4 m and g=10. Therefore, we have compared the mapping results using the best, the worst and these default values for the four datasets.

**Figure 9:**
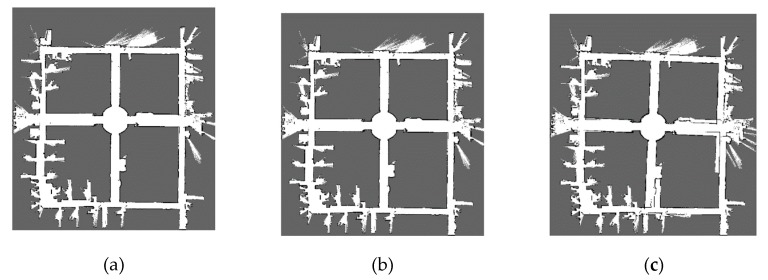
ACES dataset map results with (**a**) best, (**b**) default, (**c**) worst values.

**Figure 10:**
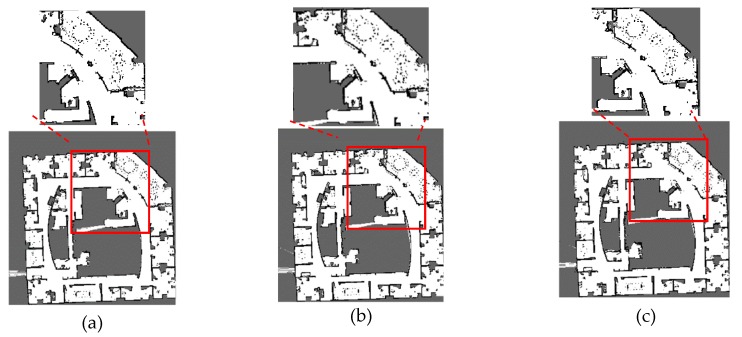
Intel dataset map results with (**a**) best, (**b**) default, (**c**) worst values.

**Figure 11:**
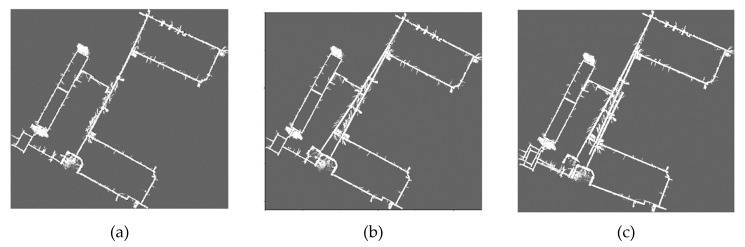
MIT-Killian dataset map results with (**a**) best, (**b**) default, (**c**) worst values.

**Figure 12:**
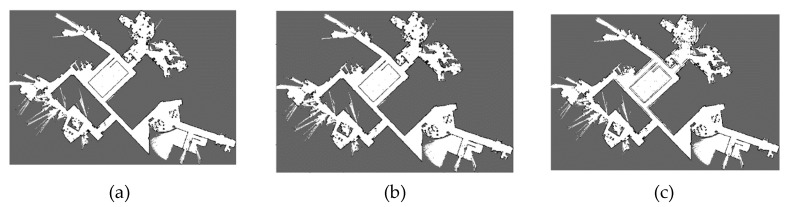
MIT-CSAIL dataset map results with (**a**) best, (**b**) default, (**c**) worst values.

The maps corresponding to these parameters of each dataset are shown in Figure 9, Figure 10, Figure 11 and Figure 12. The comparison of maps quality is intuitive to verify the effectiveness of our method. Figure 9 shows the ACES dataset map results, it can be seen from Figure 9b that the map obtained by default parameters is similar to the map of Figure 9a and the worst parameters can cause obvious wall overlaps in Figure 9c. By comparing the map results for the Intel dataset in Figure 10, we observe that the walls with the default and the worst parameters slightly overlap on the upper right portion of the maps. The map results for the MIT-Killian dataset are shown in Figure 11 and we can see large deviation error occurred in the corridor from Figure 11b,c which indicate that the back-end optimization is ineffective with the default and the worst parameters. For the MIT-CSAIL dataset, we can also see significant accumulated errors in Figure 12c with the worst parameters. Since the default parameters are close to the best parameters, their corresponding maps are similar to each other.

The three optimization objectives with the best, default and worst parameters are given in Table 4, Table 5 and Table 6 respectively. The maps obtained by Karto SLAM with the best parameters is more consistent with the real environment with lower η, fewer $n_{c}$ and fewer $n_{e}$ than the maps obtained with the worst parameters. As can be seen from Table 4 and Table 5, except for the MIT-CSAIL dataset, the values of the three metrics using the best parameters are smaller than those using the default parameters. This indicates the effectiveness of the optimal parameters compared with the default parameters. The metrics for the MIT-CSAIL dataset with the best and the default values are very close to each other. We further compare the times of global optimization with different values of r and g in Table 7. It is found that Karto SLAM performs more global optimizations with the best parameters except the Intel dataset. For the Intel dataset, the global optimization is carried out 7 times with both the best and worst parameters. A further investigation finds that five times of the optimization occurs after 10000 scans of the dataset (with a total of 13631 scans) with the best parameters while most optimization occurs in the first 10000 scans with the worst parameters, as shown in Table 8. This indicates that the global optimizations with the best values of r and g can correct more robot poses.

Also, our method has been verified using the benchmark measurement suggested in Ref. [42] in which relative relations between robot poses were extracted and every single observation between pairs of poses was validated manually. To do this, the absolute translational and rotational errors have been calculated as well as the corresponding standard deviation for each dataset, as listed in Table 9 and Table 10 respectively. It can be shown that both translational and rotational errors with the best parameters are smaller than those with the default and the worst parameters for these datasets, except for the MIT-CSAIL dataset for which the errors with the default parameters is almost identical to those with the best parameters. Again, large errors can be seen with the default and the worst parameters for the MIT-Killian dataset.

**Figure 13:**
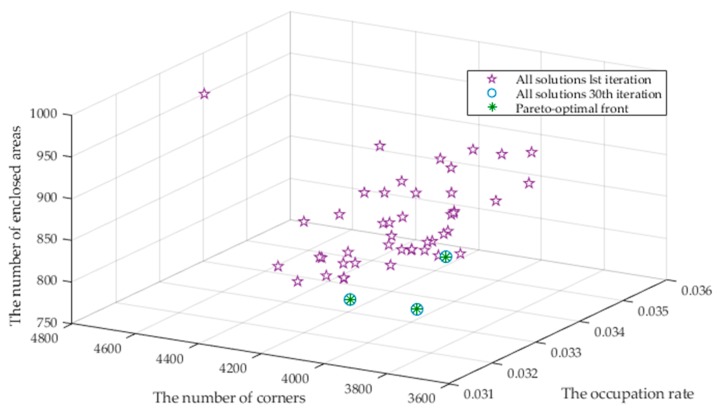
NSGA-III result for 1^st^, 30^th^ and 60^th^ iterations of ACES dataset.

Furthermore, we investigate the variations of the multi-objective solutions during the evolutionary optimization process using NSGA-III. We select the ACES dataset for demonstration. The solution distributions at the 1^st^, the 30^th^ and the last 60^th^ iterations are displayed in Figure 13. We can see that the three objectives η, $n_{c}$ and $n_{e}$ significantly decrease from the 1^st^ iteration. The results of the 30^th^ generation are very close to the Pareto optimal solution of the 60^th^ generation, which indicates that the results converge after the 30^th^ generation. A set of nondominant solutions are obtained after the multi-objective optimization with NSGA-III. In this article, we choose the solution that has the largest amount of individuals in the last generation as the final results.

**Figure 14:**
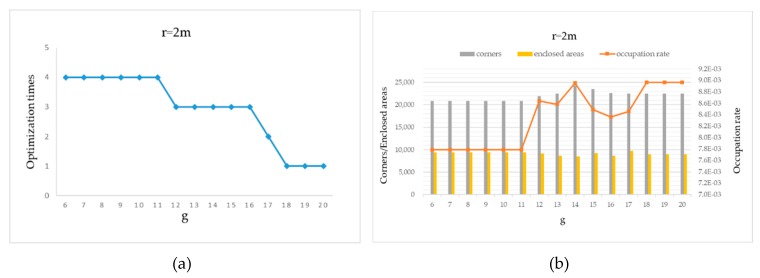
MIT-Killian dataset map results with different *g*: (**a**) optimization times and (**b**) three metrics.

**Figure 15:**
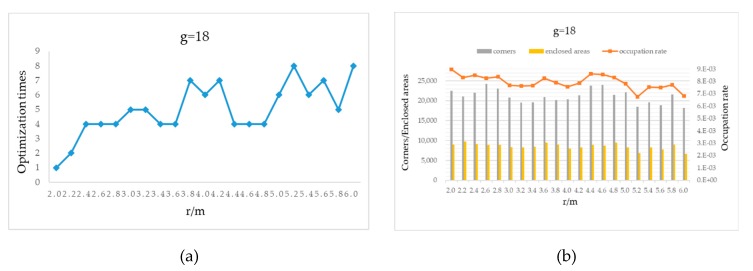
MIT-Killian dataset map results with different *r*: (**a**) optimization times and (**b**) three metrics.

We also investigate the variation of the three metrics and the optimizations times against only one parameter, while the other parameter is constant. Here the MIT-Killian dataset is used as an example for demonstration. We let r  be constant as 2 m. When g increases from 6 to 20, more nodes are required in the data chain to satisfy the loop closure condition. When g is larger than 11, the times of back-end optimization decreases as shown in Figure 14a, and the proportion of occupied grids significantly increases as shown in Figure 14b. Then we let *g* be constant as 18. As shown in Figure 15a, as the search range r increases, it becomes easier to meet the condition for loop closure and more back-end optimizations are carried out. This can be also indicated by the downward trend of the evaluation metrics in Figure 15b.

From the above results, we obtain the following observations. (1) The loop closure detection parameters can significantly affect the mapping quality. Wall overlaps, redundant corners, more enclosed areas, and even large deviation errors can occur in the maps if these parameters are not properly chosen for Karto SLAM, because loop closure detection is not successful or the times of global optimization is insufficient. (2) In the absence of ground truth, the map quality can be evaluated by the image quality in terms of the occupation rate, the number of corners and the number of enclosed areas. (3) The map quality can be improved by optimizing the loop closure detection parameters with the three evaluation metrics in Section 3.2 as the multiple objectives of NSGA-III. (4) Due to the diversity of the environments, the performance of SLAM algorithm with a single set of parameters can be different for these environments. An ideal scenario is that the SLAM parameters can be tuned and specific for each environment.

### 4.2. Real-World Experimental Results

**Figure 16:**
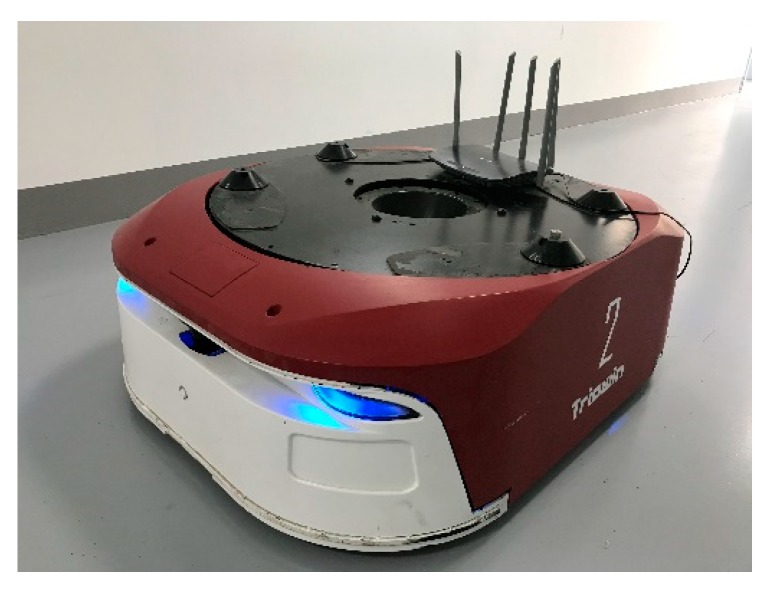
TRIOWIN mobile robot with mounted sensors.

Real-world experiments have been conducted to validate the proposed method for the tuning of loop closure detection parameters. For this purpose, a TRIOWIN mobile robot (see Figure 16) controlled by a PC and equipped with two SICK TIM571 laser sensors was used in the experiments. The laser system operates at 15 Hz, with a scanning angle range of 270°, an angular resolution of 0.33°, and a maximum effective range of 25 meters. We chose the long distance (70-meter) corridor on Floor 2, Block A, Building 8 and the underground garage on Floor B1 in the same building at the Shanghai Electrical Apparatus Research Institute (SEARI) as our test environments. For both experiments, the mobile robot moved back and forth twice to verify whether Karto SLAM can detect the loop closure and trigger the back-end global optimization. In the experiments, we set p=0.7 for the long corridor environment p=0.4 for the underground environment.

**Figure 17:**
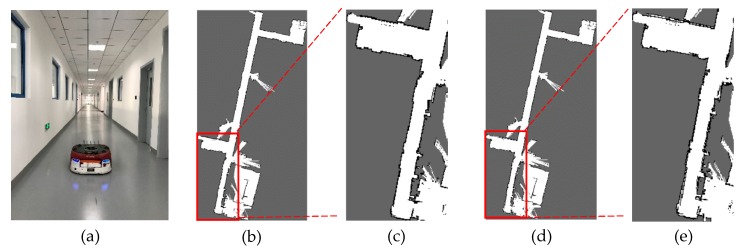
(**a**) Actual environment of long corridor in SEARI- Floor 2, Block A, Building 8 and map results with (**b**) best, (**c**) is a partially enlarged view of (b), (**d**) worst values. and (**e**) is a partially enlarged view of (d).

**Figure 18:**
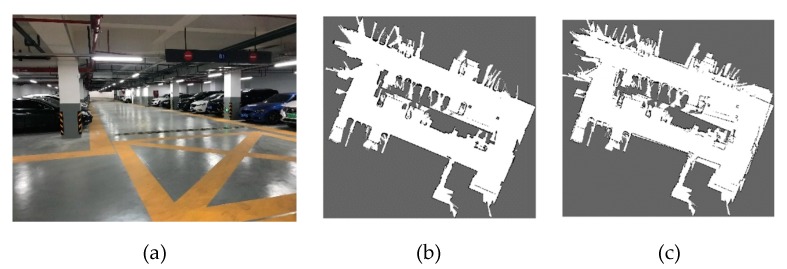
(**a**) Actual environment of underground garage in SEARI- Floor B1, Block A, Building 8 and map results with (**b**) best, (**c**) worst values.

The best and worst values of r and g obtained through NSGA-III are listed in Table 11 for the two environments, respectively. Figure 17 shows the environment and the map results of the 70-meter long corridor on Floor 2, Block A, Building 8, SEARI. The maps constructed with the best and the worst parameters are shown in Figure 17b–e respectively. We can see that the wall can slightly overlap if the worst loop closure detection parameters are used. Figure 18 shows the environment and the map results of the garage of about 4,000 square meters on Floor B1, Block A, Building 8, SEARI. The maps constructed with the best and the worst parameters are shown in Figure 18b,c, respectively. It can be observed that the map obtained from the best parameters is more consistent with the real environment. Obvious wall overlaps and deviation errors can be generated if the loop closure detection parameters are not chosen properly.

The optimization objectives, i.e., the occupation rate, the number of corners and the number of enclosed areas, are given in Table 12 and Table 13 with different parameters respectively. These results indicate that the optimal loop closure detection parameters lead to better mapping quality especially for the large underground garage environment. If the parameters are not properly chosen, the maps become blurrier and have overlapping walls, redundant corners, and more enclosed areas. This is because the maps built in the second cycle is not aligned with those built in the first cycle and thus experience deviation, which can be proved by comparing the times of global optimizations with different parameters as listed in Table 14. If the loop closure detection parameters are not chosen properly, the SLAM algorithm performs the back-end optimization only once for the two environments, and thus accumulates considerable errors especially for the large underground garage environment.

**Figure 19:**
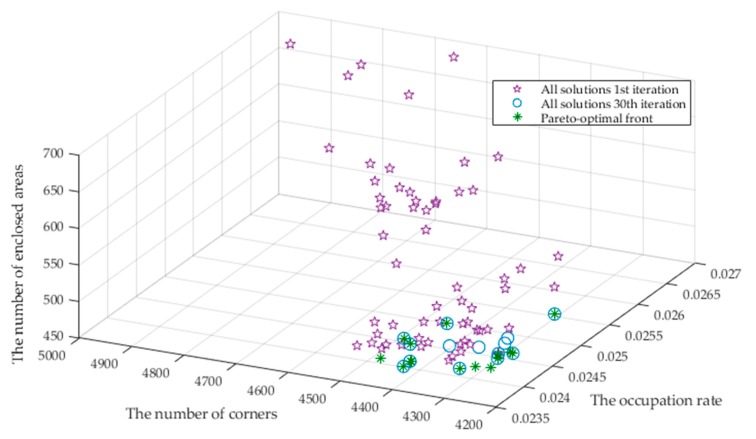
NSGA-III result for 1^st^, 30^th^ and 60^th^ iterations of the underground garage.

We further use the underground garage as an example to demonstrate the variations of the three objectives during the NSGA-III optimization in Figure 19. Similar to the results for the datasets, the three objectives η, $n_{c}$ and $n_{e}$ significantly decrease from the 1^st^ iteration. The results of the 30^th^ generation tend to be consistent with the Pareto optimal solution of the 60^th^ generation, which indicates the convergence of the multi-objective optimization. 

More importantly, Karto SLAM, the computation of the three evaluation metrics and the NSGA-III algorithm are all implemented on a computer. As a result, the loop closure detection parameter optimization and the corresponding mapping can be completed without human intervention. 

## 5. Conclusions

A multi-objective optimization method is proposed for the loop closure detection parameters for indoor 2D graph-based SLAM. The method integrates the Karto SLAM algorithm, an evaluation approach for map quality with three metrics in the absence of ground truth, and the NSGA-III multi-objective optimization algorithm. The two loop closure detection parameters under study represent the condition for whether the SLAM algorithm should trigger the back-end optimization of graph-based SLAM. The three optimization objectives, i.e., the occupation rate, the number of corners, and the number of enclosed areas, reflect the deviation errors such as overlaps, blurring and misalignment when mapping multiple nested loops. The proposed method has been implemented and validated by testing on four datasets and two real-world environments. 

It has been demonstrated that wall overlaps, redundant corners, more enclosed areas, and even large deviation errors can occur in the maps if the parameters are not properly tuned for Karto SLAM. For all the tests, the map quality can be improved by optimizing the loop closure detection parameters with the three evaluation metrics as the multiple objectives of NSGA-III, which can be proved by the iterative results of the optimization objectives or by the observation on the images. The proposed optimization method for loop closure detection parameters and the corresponding map improvement can be completed by running NSGA-III algorithm without human intervention, which provides potential applications for automatic tuning of these parameters for indoor 2D SLAM to obtain a high-quality map without ground truth.

In this work, we find that the NSGA-III algorithm requires a considerable computational time for the SLAM parameter optimization with the three objectives. Therefore, in future research, we will investigate how to improve the efficiency of the proposed method and whether it is possible to reduce the number optimization objectives. We will also further analyze the influence of other parameters in SLAM algorithms on the mapping quality and the possibility of optimizing other parameters using the proposed multi-objective optimization method. 

## Figures and Tables

**Table 1:** Summary of map evaluation methods.

Evaluation Methods	Refs and Publication Years	Pros and Cons
Ground truth map	[31] (2013); [32] (2008); [33] (2008); [34] (2008); [35] (2013) [36] (2015); [37] (2010)	Accurate by direct comparison with the ground truth map; but difficult to obtain the ground truth map.
Ground truth trajectory	[38] (2015); [39] (2017); [40] (2007); [41] (2009); [42] (2009)	Easier to obtain ground truth tracjectory than ground truth map, suitable for large scale environments; but still requiring manually calibration of the groud truth trajectory.
No ground truth	[43] (2008); [44] (2017)	No need for ground truth, better applicability; but less accurate than the comparison with ground truth.

**Table 2:** The parametric description and values used in NSGA-III for the four datasets.

Parameters	Description	Values
r	Search radius	With a range of [2,6] m
g	Size of chain	With a range of [6,20]
q	Diagonal terms in position covariance matrix	0.16
p	Response value	0.7
M	Number of objectives	3
D	Divisions of each objective axis	10
Gmax	Maximum number of iterations	60
N	Population size	66

**Table 3:** The parametric results of *r* and *g* with NSGA-III for datasets.

	ACES	Intel	MIT-Killian	MIT-CSAIL
Best	(5.29 m,17 size)	(2.27 m,19 size)	(5.05 m,11 size)	(4.02 m,10 size)
Worst	(2.16 m,18 size)	(3.28 m,16 size)	(2 m,18 size)	(2.21 m,20 size)

**Table 4:** Results of three objectives with best parameters for datasets.

Indicators	ACES	Intel	MIT-Killian	MIT-CSAIL
η	0.0320	0.0778	0.0067	0.0344
$n_{c}$	3837	3721	18297	4957
$n_{e}$	801	344	6737	687

**Table 5:** Results of three objectives with default parameters for datasets.

Indicators	ACES	Intel	MIT-Killian	MIT-CSAIL
η	0.0329	0.0805	0.0075	0.0344
$n_{c}$	4167	3732	20330	4957
$n_{e}$	862	400	7988	687

**Table 6:** Results of three objectives with worst parameters for datasets.

Indicators	ACES	Intel	MIT-Killian	MIT-CSAIL
η	0.0341	0.0834	0.0089	0.0379
$n_{c}$	4705	3783	22507	5316
$n_{e}$	957	429	8965	1084

**Table 7:** Optimization times with different parameters for datasets.

Optimization Times	ACES	Intel	MIT-Killian	MIT-CSAIL
Best	4	7	9	3
Default	3	6	6	3
Worst	1	7	1	0

**Table 8:** The location of optimization for the Intel dataset.

	1	2	3	4	5	6	7
Best	1951	6745	12122	12738	13203	13274	13314
Worst	1853	1911	2264	8140	12722	13274	13400

**Table 9:** Results of absolute translational errors with different parameters for datasets.

Absolute Translational $\mathrm{Error}{/m}^{2}$	ACES	Intel	MIT-Killian	MIT-CSAIL
Best	0.0608 ± 0.0668	0.0616 ± 0.06741	0.0367 ± 0.0318	0.0369 ± 0.0348
Default	0.0621 ± 0.0759	0.0772 ± 0.11466	0.545 ± 2.4180	0.0369 ± 0.0348
Worst	0.1035 ± 0.2657	0.0766 ± 0.1110	1.2259 ± 3.3468	0.0889 ± 0.1847

**Table 10:** Results of absolute rotational errors with different parameters for datasets.

Absolute Rotational $\mathrm{Error}{/\deg}^{2}$	ACES	Intel	MIT-Killian	MIT-CSAIL
Best	0.0159 ± 0.0205	0.0249 ± 0.0431	0.0067 ± 0.0101	0.0190 ± 0.0333
Default	0.0159 ± 0.0207	0.0253 ± 0.0429	0.0159 ± 0.0218	0.0190 ± 0.0333
Worst	0.0162 ± 0.0208	0.0253 ± 0.0429	0.0167 ± 0.0294	0.0191 ± 0.0334

**Table 11:** The parametric results of *r* and *g* of NSGA-III iteration for real-world tests.

	Long Distance Corridor	Underground Garage
Best	(3.13 m,6 size)	(5.51 m,16 size)
Worst	(3.34 m,13 size)	(2.19 m,19 size)

**Table 12:** Results of three optimization objectives with best parameters for real environments.

Indicators	Long Distance Corridor	Underground Garage
η	0.0176	0.0241
$n_{c}$	1641	4259
$n_{e}$	259	478

**Table 13:** Results of three optimization objectives with worst parameters for real environments.

Indicators	Long Distance Corridor	Underground Garage
η	0.0189	0.0268
$n_{c}$	1707	5005
$n_{e}$	280	714

**Table 14:** Optimization times for real environments with different parameters.

	Long Distance Corridor	Underground Garage
Best	2	3
Worst	1	1
